# Management of Laryngotracheal Trauma: A Five-Year Single Institution Experience

**Published:** 2018-09

**Authors:** Pradipta-Kumar Parida, Raja Kalaiarasi, Arun Alexander

**Affiliations:** 1 *Department of ENT and Head Neck Surgery, AIIMS Bhubaneswar, Bhubaneswar-751019, Odisha, India.*; 2 *Department of ENT, Sri Lakshmi Narayana Institute of Medical Sciences, Pondicherry- 09,* *Pondicherry, India.*; 3 *Department* * of ENT and Head Neck Surgery, Jawaharlal Institute of Postgraduate Medical Education & Research (JIPMER), Pondicherry, * *India.*

**Keywords:** Arytenoid cartilage, Larynx, Laryngeal cartilages, Neck injury

## Abstract

**Introduction:**

Laryngotracheal trauma is a rare life-threatening emergency that requires early identification and immediate intervention. Here, we present 26 patients with laryngotracheal trauma from a tertiary hospital in India. The aim was to describe the clinical presentation and management of laryngotracheal trauma patients.

**Materials and Methods:**

This was a retrospective study of laryngotracheal trauma patients treated between January 2011 and March 2016. Patients who presented with a breach in the laryngotracheal framework were included, while those who had penetrating neck injuries superficial to strap muscles/platysma, burn injuries, caustic ingestion, or endotracheal injuries were excluded from the study.

**Results:**

Of 253 patients with neck injury, 26 (23 adults, three children; 21 males, five females; age range, 5-60 years) presented with a breach in the laryngotracheal framework (15 blunt neck-trauma patients and 11 penetrating neck-trauma patients). The most common cause of neck injury was road traffic accidents, seen in 12 patients (46.2%). Computed tomography (CT) was performed in all blunt trauma cases and in four patients with penetrating trauma. All penetrating trauma patients underwent neck exploration. Twelve blunt trauma patients (46.1%) were managed conservatively, while three (11.5%) required surgical intervention. The most common neck exploration finding noted in patients with a penetrating injury was fracture of the thyroid cartilage, which was seen in eight patients (30.8%). Twenty patients (76.9%) had a normal voice, five patients (19.2%) had a hoarse voice, and one patient (3.8%) had a breathy voice post treatment.

**Conclusion:**

Early intervention of laryngotracheal trauma is crucial. The role of a CT scan is essential in decision making in blunt trauma cases.

## Introduction

Laryngotracheal (LT) injuries are a rare but life-threatening emergency. LT trauma constitutes less than 1% of all trauma cases, but accounts for more than 75% cases of immediate mortality (1). While the incidence of LT injury is 1 in 30,000 emergency admissions in the United States (2,3), the exact incidence in India is not known due to the paucity of large serial studies published on neck trauma. LT injuries are less common in the pediatric population because of the higher position and elastic nature of the larynx (4). The vital structures involved in ventilation, phonation, and swallowing are present in a more compact space in the neck and are more prone to injury as they are not protected by a bony covering.

LT trauma may also be associated with other organ injuries. The role of the otolaryngologist in identifying airway emergencies and securing the airway patency should be the first priority in multi-organ injuries. An organized and multidisciplinary approach to trauma cases is required to prevent misdiagnosis and inadequate management. Neck injuries are classified either as blunt or penetrating trauma; blunt trauma cases involve tissue disruption, while tissue loss occurs in penetrating injuries. Injury to the larynx may range from minor mucosal edema to displaced fracture of the laryngeal cartilage framework.

Many controversies exist in the management of neck injury, such as whether the type and mechanism of injury should direct the management approach. One area that particularly lacks clear guidance is therapeutic decision making, including whether to mandatorily or selectively explore the neck. However, advances in imaging studies have provided a valuable tool for treatment planning in trauma cases. The restoration of phonatory and the sphincteric function of the larynx depends on the proper alignment of the cartilaginous framework, muscles, ligaments, as well as mucosal integrity following injury. The early detection and management of such a rare challenging entity is very important for an otolaryngologist. The aim of this study was to review the data of LT trauma patients in our hospital.

This study is reported from our tertiary health care center in South India which is located on a national highway, and where we commonly encounter neck injuries of various causes. The incidence of neck trauma reported at our center is approximately 50 patients per year, of whom an average of five patients present with LT-framework involvement. The aims of this study were to describe the clinical presentation, management, and outcomes in patients who presented with LT trauma over a 5-year period.

## Materials and Methods

A retrospective chart review of patients hospitalized with LT injuries at a tertiary hospital in South India between January 2011 and March 2016 was carried out. A total of 253 patients presented with neck injury. Patients who had penetrating neck injuries superficial to platysma/strap muscles, burn injuries, caustic ingestion, or endotracheal injuries were excluded from the study. Of 253 patients, 26 patients who presented with a breach in the LT framework (identified by clinical examination and by radiological study) were included in the study. Patient demographics such as age, sex, mode of injury, time to reach the tertiary center, clinical presentation, site, and level of injury were recorded. Patients underwent a thorough physical examination, endoscopic examination of the larynx, radiographic investigations, and management for their condition.Continuous variables including age were tested for normality using the Kolmogorov–Smirnov (K-S) test and were reported as mean ± standard deviation (SD) or median. Categorical variables including gender, type of injury, clinical presentation, and laryngeal findings were reported as percentages or proportions. The analysis was performed using Microsoft Excel and SPSS version 17.

## Results

*Demographic data*

Twenty-six patients were admitted with LT trauma, of whom 21 were male and five were female (male:female ratio, 4.2:1), revealing a notable male preponderance. In total, 23 adult and three pediatric patients, ranging in age from 5 to 60 years, were included in the study. The mean age of the adult and pediatric patients were 37.3±2.3 years and 7.4±1.5 years, respectively. Fifteen patients (57.7%) sustained blunt neck trauma and 11 were admitted with penetrating neck injury (Table.1).

**Table 1:** Type of injury

**Subject No.**	**Type** ** of injury** **(N, %)**	**Etiology**	**patients ** **N (%) **
1	Blunt trauma (15, 57.7%)	Road traffic accident	12(46.2%)
		Hanging (clothesline injury)	3(11.5%)
2	Penetrating injury (11, 42.3%)	Cut throat (homicidal)	5(19.3%)
		Cut throat (suicidal)	4(15.4%)
		Bull-gore injury	1(3.8%)
		Pen nib injury	1(3.8%)

The most common cause of blunt trauma was road traffic accidents, which was seen in 12 patients (46.2%). The leading causes of LT injuries were homicidal and suicidal attempts in females. Only eight patients (30.8%) reached the tertiary center within 6 hours of injury, while 18 patients (69.2%) arrived after 6 hours. Patients who presented late received first aid in the nearby hospital. The average time elapsed between the event and the surgical intervention was 9 hours (range, 4–24 hours; median, 8 hours). Three patients arrived at the hospital already intubated with an endotracheal tube.

*Clinical presentation*

The most common symptom at the time of the initial assessment was respiratory distress in the form of breathing difficulties and stridor, which was seen in 18 patients (69.2%), followed by subcutaneous emphysema (61.5%) (Table.2).

**Table 2:** Clinical presentation of the patients at the time of initial assessment

**Subject No.**	**Clinical presentation**	**N (%)**
1	Respiratory distress	18 (69.2%)
2	Surgical emphysema	16 (61.5%)
3	Dysphonia	8 (30.8%)
4	Bleeding from the site	6 (23.1%)
5	Dysphagia	4 (15.4%)
6	Hemoptysis	4 (15.4%)
7	Aphonia	3 (11.5%)
8	Endotracheal tube in situ	3 (11.5%)

All neck injury patients were evaluated by the emergency trauma team, and five patients (19.2%) were found to have other associated injuries. Hypopharyngeal and esophageal injury was seen in two patients (7.7%), while brain contusion and fracture of the angle of the mandible was seen in one patient (3.8%), and incomplete facial nerve palsy was seen in one patient (3.8%). All patients underwent fiberoptic flexible endoscopic examination, with the most common laryngeal finding being congested and edematous vocal cord seen in 24 (92.3%) patients (Table.3).

**Table 3:** Endoscopic/ direct laryngoscopic assessment findings in patients with Laryngotracheal trauma

**Subject** ** No.**	**Laryngeal findings**	**N (%)**
1	Congestion and edematous vocal cord	24 (92.3%)
2	Hematoma of the vocal cord	14 (53.8%)
3	Restricted vocal cord mobility	4 (15.4%)
4	Unilateral vocal cord palsy	2 (7.7%)
5	Avulsed left cord	1 (3.8%)
6	Hematoma of aryepiglottic fold & avulsed epiglottis	1 (3.8%)
7	Avulsed anterior commissure	1 (3.8%)

*Radiological investigations*

All neck-trauma patients underwent anterior neck and chest radiograph. Contrast-enhanced computed tomography (CECT) of the neck was performed in all 15 blunt trauma patients (57.7%) and in four patients (15.4%) with a penetrating neck injury. CECT imaging was not performed in seven patients (26.9%) with an obvious open-neck wounds who clearly required neck exploration. All patients showed dissection of air in the subcutaneous plane. Ten patients (38.5%) showed thyroid cartilage fracture, of whom, four (15.3%) had a displaced fracture. Seven blunt trauma patients (27%) showed an isolated tracheal injury, while one patient (3.8%) showed cricoid cartilage fracture with dislocation of the cricoarytenoid joint. Postoperative chest radiographs were performed in all patients who underwent neck exploration. Gastrografin swallow was performed in two blunt trauma patients who had odynodysphagia and were suspected to have esophageal injury. None of the patients had any lung or pleural injury.

*Treatment of LT injuries and associated complications*

All patients with penetrating neck injuries underwent neck exploration. Major vessel involvement was seen only in one patient (3.8%), who had laceration of the internal jugular vein leading to massive blood loss. The remaining 25 patients (96.1%) had intact major vessels. Twelve patients (46.2%) were managed conservatively and 14 patients (53.8%) received surgical intervention. Conservative management included elevation of the head end to 30 degrees, administration of oxygen by mask, voice rest, intravenous broad-spectrum antibiotics, intravenous followed by oral steroids, and antireflux medications such as proton-pump inhibitors. Blunt injury patients who had severe respiratory distress, worsening of symptoms in terms of extending subcutaneous emphysema on conservative management and radiological evidence of dislocated arytenoid cartilage, major endolaryngeal laceration, or evidence of compromised airway underwent neck exploration. One patient who had a severely avulsed left vocal cord, underwent CO_2_ laser cordotomy. Blunt trauma patients who had minor injuries were managed conservatively. In two blunt trauma patients who developed significant stridor due to airway edema defunctioning tracheostomy was performed without LT repair (Table.4).

**Table 4:** Treatment outline of the patients with laryngotracheal injury

**Subject No.**	**Treatment given**	**Type of injury**	**N (%)**
1	Conservative management	Blunt trauma	12 (46.2%)
2	Tracheostomy and primary LT repair	Penetrating trauma	9 (34.6%)
3	Tracheostomy followed by close observation	Blunt trauma	2 (7.7%)
4	Neck exploration without tracheostomy	Penetrating trauma (isolated tracheal injury)	2 (7.7%)
5	CO_2_ laser excision of severely avulsed cord with tracheostomy	Blunt trauma	1 (3.8%)

All pediatric blunt trauma patients (7.7%) were managed conservatively. All 12 tracheostomized patients (46.1%) were decannulated successfully within 3 months. Five patients (19.2%) had mild dysphonia following treatment for LT injury (Table.5).

**Table 5:** Complications following laryngotracheal trauma treatment

**Subject No.**	**Complications**	**N (%)**
1.	Mild dysphonia	5(19.2%)
2.	Aspiration	3(11.5%)
3.	Wound infection	3(11.5%)
4.	Granulation tissue	1(3.8%)
5.	Residual vocal cord palsy	1(3.8%)
6.	Grade 1 subglottic stenosis	1(3.8%)
7.	Poor (breathy) voice	1(3.8%)

One patient (3.8%) who underwent anterior commissure repair developed granulation tissue following the repair and required a second procedure. In that case, the granulation tissues were removed using a laryngeal microdebrider. Two patients (7.7%) had associated hypopharyngeal and esophageal injuries, which were sutured primarily during laryngeal repair. One patient (3.8%) had a fracture of the angle of the mandible and was managed with inter-maxillary fixation wiring for 4 weeks. One patient (3.8%) with mild brain contusion with no mid-line shift on CECT brain imaging was managed conservatively with head-end elevation. One patient (3.8%) with incomplete facial nerve palsy was managed with oral steroids, eye care, and physiotherapy.

The average duration of hospital stay was 10 days (range 4–14 days). Among the 12 patients (46.2%) who required no surgical intervention, the average hospital stay was 7 days, while patients who underwent laryngeal repair had an average hospital stay of 12 days. During 1 month of follow up, a flexible endoscopic assessment was performed on all patients. Patients were followed up after 3 months and 6 months following discharge from the hospital. Seven patients (26.93%) were lost to follow up at 3 and 6 months.

## Discussion

Otolaryngologists play a significant role in the successful management of LT injury. The incidence of LT injuries varies from one region to another. In our study, young adults were found to have more LT injuries than older people because they are involved more frequently in road traffic accidents. In addition, males sustain LT injury more commonly than females because of their greater participation in violent sports and activities such as fighting. A similar demographic distribution was observed in other studies (5,6).

In our study, blunt LT injuries were more common than penetrating injuries. This is in contrast with a study by Sachdeva et al., who observed penetrating neck injury more commonly than blunt trauma (6). The type of injury depends on the mode of injury, nature of the object that cause the injury, location and velocity of the impact force, and patient-related factors (such as age and ossification of the laryngeal cartilages) which can result in minor injury to fracture of the laryngeal cartilage, cricothyroid or cricotracheal separation associated with recurrent laryngeal nerve damage.

Sabbir et al. observed that cut-throat neck injuries were more common than road traffic accidents in their study (7). In our study, the most common cause of LT injury was motor vehicle accidents followed by homicidal cut-throat injuries. Road traffic accident neck injuries were common in our area because of a lack of awareness among the general population regarding the use of seat belts. Penetrating injuries were commonly located over or below the thyroid cartilage, with rupture of the cricothyroid or thyrohyoid which are weak regions in the laryngeal framework. Most of the penetrating injuries were horizontal or oblique deeper cuts resulting in an open wound. Most of the patients were referred to the tertiary hospital for appropriate intervention within 24 hours. The extent and depth of the injuries are best assessed within 24 hours of the accident.

In our study, the majority of the neck injury patients presented with respiratory distress and subcutaneous emphysema. Studies by Schaefer et al., Yen et al., and Cherian et al. also found respiratory symptoms as the most common presentation of neck injury, followed by hoarseness of voice, neck tenderness, and subcutaneous emphysema (2,8,9). Stridor, subcutaneous emphysema, neck tenderness, dysphagia, and hemoptysis are the clinical red signs of severe laryngeal injury (7).

The initial assessment should include a general survey for evidence of any other organ injury. In our study, LT injuries were associated with other injuries in five patients (19.2%). In some cases, subtle respiratory symptoms go unidentified due to associated injuries (10). Therefore, all multi-trauma patients need to be assessed by a team of specialists. In our study, pharyngeal and esophageal injuries were seen with penetrating injury. Bedside gentle flexible laryngoscopic assessment was attempted in all neck-trauma patients to assess the extent of the endolaryngeal injury. This should be performed on all airway-stable and hemodynamically stable patients. The most common endoscopic laryngeal findings were congested and edematous cords, which were seen in 24 patients (92.3%), indicating loose mucosal attachment and potential spaces in the larynx.

A chest radiograph is an important initial study to rule out pneumothorax or pneumomediastinum, while a CT scan helps to identify the underlying laryngeal or pharyngeal injuries (11,12). In our study, CT scan was undertaken in all blunt trauma patients and in selected patients with penetrating injury. In blunt trauma patients, CT scan will help in differentiating patients who can be managed conservatively versus those who require neck exploration (13). Blunt trauma patients who showed radiological red signs, such as evidence of cartilage exposure, arytenoid dislocation, displaced cartilage fracture, vocal cord immobility or tear, or major endolaryngeal hematoma with impending airway compromise on CT scan underwent neck exploration. CT scan is not indicated in patients with an open wound with obvious fracture of the laryngeal cartilages who clearly need surgical intervention. Flexible endoscopy combined with a CT scan is a reliable tool in the evaluation of injured larynx that lacks definitive indications for neck exploration, avoiding negative explorations and providing guidance on appropriate management (13).

Gastrografin swallow studies are indicated in patients on conservative management in whom pharyngeal or esophageal injury is suspected. Gastrografin is preferred over barium as it is less irritating to body tissues if there is a leak from the pharynx or esophagus (14). Treatment should be individualized based on the type and severity of the injury. Direct laryngoscopy to assess the larynx and rigid esophagoscopy to assess for any pharyngeal and upper esophageal injury should precede the neck exploration (15). In an intubated patient, direct laryngoscopy was performed after ruling out cervical spine injury. Endotracheal tube was retrieved to inspect the supraglottis, glottis, and subglottis for traumatic lesions using a Hopkins 0-degree long endoscope. In all patients with an open wound, the neck was explored through the neck wound itself, using the path already created by the cutting instrument to identify the depth and extent of the injury. In our study, the most common laryngeal injury found was a fracture of the thyroid cartilage. All displaced and comminuted fractures were reduced and fixed with nonabsorbable 2-0 PROLENE. Other authors used Mini-plates to fix the displaced fracture (11,16). 

**Fig 1:**
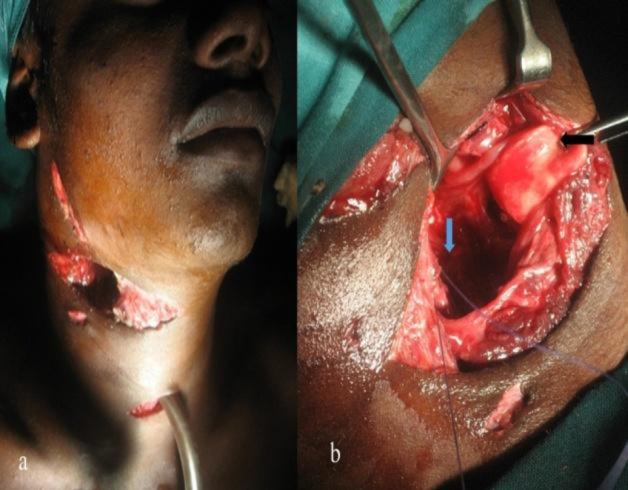
a) Patient with bullgore injury in the lateral neck. b) Intraoperative field showing avulsed epiglottis (black arrow) and opened up pharyngeal space (blue arrow).

In one patient who had a bull-gore injury, there was significant tissue loss and avulsed epiglottis with associated pharyngeal injury (Fig.1) which was managed by suturing the epiglottis to the external intact perichondrium of the thyroid cartilage with pharyngeal repair.

**Fig 2:**
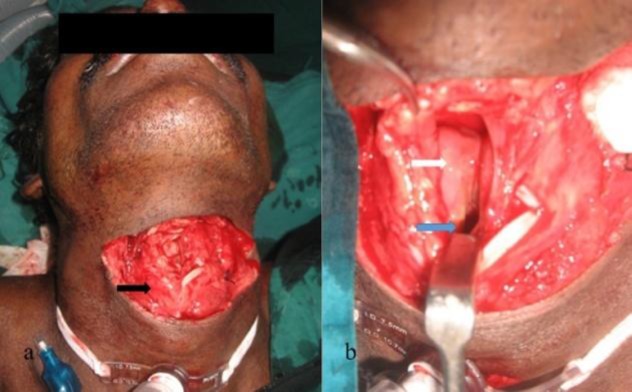
a) Penetrating neck injury with the plane of transection passing through thyrohyoid membrane level opening the supraglottis with tilted thyroid cartilage (black arrow). b) Open wound showing vocal cord (blue arrow) with oedematous arytenoids (white arrow).

One patient who had severe supraglottic injury with fractures of the thyroid cartilage associated with bulky arytenoid (Fig.2) had delayed wound healing. 

Generally, patients who had supraglottic injury had delayed wound healing and delayed decannulation. Arytenoid dislocation indicates major laryngeal injury that was seen in one patient who was managed by reducing the displacement but failed to regain cord mobility. Stanley et al. reported that arytenoid dislocation is a poor prognostic sign with respect to cord mobility and voice quality (17). Other authors also drew similar conclusions regarding LT injuries with arytenoid dislocation (18–20).

**Fig 3:**
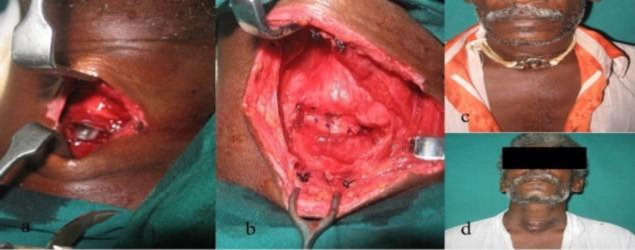
a) Clinical photograph of a patient with penetrating neck injury showing partial cricotracheal transection with endotracheal tube insitu. b) Surgical field showing approximated cricoid and tracheal segment with 2-0 prolene. c) Patient at 1 month follow up after discharge.d) Patient after decannulation.

In 12 patients (46.1%), tracheostomy was performed under local anesthesia followed by administration of general anesthesia through the tracheostomy tube for LT assessment/ reconstruction. One patient had a partial cricotracheal transection and came to us with endolaryngeal tube in situ (Fig.3).

Although oral endotracheal intubation is not contraindicated, it is better avoided as it can result in further disruption of the endolarynx and false passage formation. Three patients were referred to us with endotracheal tube *in situ*; fortunately, none of these experienced have any of these complications.

Laryngeal/pharyngeal mucosal lacerations were closed after redraping of the mucosa over the exposed cartilage. In blunt trauma, the clinical status of the patients was closely monitored by frequent examination, whether subcutaneous emphysema was extending or reducing. Nonsurgical management was limited to patients who had minor mucosal injuries without airway compromise. Recent guidelines on the management of adult penetrating neck trauma by the Western Trauma Association recommend non-operative management in patients without dysphagia, hoarseness, hemoptysis, hematemesis, abnormal x-ray findings, or bruits/thrills (21).

LT injuries are very rare in the pediatric population due to the high position of the larynx in the neck, the pliable nature of the cartilages and due to the fact that the larynx is well protected by the mandible (22). In adults, road traffic accidents, assaults, and suicide attempts are the most common causes of external laryngeal injuries, while in children, play activities such as cycling and accidental falls are the most common causes of LT injury. In our study, out of three pediatric patients (11.5%), two (7.7%) had blunt trauma. Blunt laryngeal trauma generally occurs when the neck is hyperextended. Penetrating injuries in children are extremely rare and usually occur with cutting instruments (23). One pediatric patient had an accidental tracheal injury with a pen tip which pierced the anterior tracheal wall. He was managed with neck exploration and the rent in the fourth tracheal ring was closed with a Vicryl 3-0 suture. Simple tracheal lacerations without detached tracheal ring were repaired without a tracheostomy. Clinically stable pediatric patients with penetrating neck injury can be managed conservatively without immediate exploration (24,25).

Follow-up of the patient until complete restoration of the normal voice and resolution of the subcutaneous emphysema is mandatory. The time delay before surgical exploration is an important prognostic indicator. Delayed repair may result in higher chances of laryngeal stenosis, scarring, and granulation tissue formation. Decannulation was performed successfully in all patients within 3 months. At 1-year follow-up, one patient (3.8%) had restriction of cord mobility. All patients had functional voice, with mild hoarseness in five patients (19.2%), and all had near normal deglutition. Jalisi et al. found that all patients in their study had a good voice quality, and all tracheostomized patients were decannulated successfully (26). The major limitation of this study was loss of patients for long-term follow up, meaning that late complications such as stenosis of the airway or quality of voice could not be studied.

## Conclusion

According to our study, we recommend meticulous clinical examination supported with CECT imaging study in blunt trauma patients. In blunt trauma, symptoms and signs may hide underlying severe laryngeal injury. The role of the CT scan is crucial in decision making in blunt trauma cases. If imaging shows red signs such as evidence of cartilage exposure, arytenoid dislocation, displaced cartilage fracture, vocal cord immobility or tear, or major endolaryngeal hematoma, neck exploration is mandated. Minor endolaryngeal injuries have good outcomes with conservative management. Unfavorable outcomes are more likely with more severe laryngeal injuries. The airway must be secured first, and the preferred way is by carrying out a tracheostomy. Meticulous and early repair of the laryngeal mucosa, pharynx, esophagus, and bleeding vessels should be performed in layer closure to prevent serious complications such as LT stenosis, dysphonia, wound dehiscence, granulation tissue and fistula formation. A multidisciplinary approach is required in trauma patients to identify other co-existing injuries. Timely intervention plays a vital role in providing the best functional outcome. Large-scale studies with a longer follow-up period are warranted to develop a management algorithm.
